# Acute gallbladder perforation with gallstones spillage in a cirrhotic patient

**DOI:** 10.1186/1749-7922-5-11

**Published:** 2010-04-25

**Authors:** Costanza Chiapponi, Stephan Wirth, Matthias Siebeck

**Affiliations:** 1 Department of Surgery, Hospital of the Ludwig-Maximilians-University, Nussbaumstr. 20, 80336 Munich, Germany; 2 Department of Clinical Radiology, Hospital of the Ludwig-Maximilians-University, Ziemssenstr. 1, 80336 Munich, Germany

## Abstract

Gallbladder perforation is a rare complication of cholecystitis and cholelithiasis. The high morbidity and mortality rates associated with this condition are due to delays in diagnosis and treatment since signs and symptoms of perforation do not differ significantly from those of uncomplicated cholecystitis. We report on a patient who was affected by Child-Pugh A alcoholic liver cirrhosis and who developed an acute gallbladder perforation with spillage of stones into the peritoneal cavity and give a review of the current literature.

## Introduction

Asymptomatic cholelithiasis is a frequent condition which affects up to 10% of the adult population in wealthy nations. Acute cholecystitis develops in up to 2% of patients affected by asymptomatic cholelithiasis. Gallbladder perforation occurs in 2 to 11% of acute cholecystitis cases. Due to the high mortality that can be caused by a delay in the correct diagnosis and following adequate surgical treatment, gallbladder perforation represents a special diagnostic and surgical challenge [1].

According to Niemeier (1934), perforations are classified into three categories: type I includes patients with free perforation into the peritoneal cavity, type II describes patients with localized perforation and type III patients with cholecysto-enteric fistulas. Less frequent forms include cholecystobiliary fistula and more complex fistula formations [2]. Cases of intrahepatic perforation of the gallbladder with liver abscess and cholecystohepatic communication have also been reported [3].

## Case Report

A 49-year-old man with liver cirrhosis and a history of esophagial varices presented to a clinic with upper abdominal pain. He described colicky pain radiating to the back. He denied nausea, vomiting, diarrhea or obstipation. There was no history of gallbladder disease, no prior episode of abdominal discomfort, no medication - especially no NSAIDs - and no history of trauma. A distended abdomen with normal bowel sounds, tenderness in the right upper quadrant and signs of beginning peritoneal irritation were present. The laboratory studies showed a slightly elevated white cell count (12 G/L). All other findings were within the normal limits, including lipase and amylase, bilirubin, liver enzymes and coagulation parameters. Sonography revealed no abnormalities but failed to visualize the gallbladder. Gastroscopy confirmed the presence of type I esophageal varices. No signs of gastritis and no ulcers were reported. Computed tomography of the abdomen revealed several calcified stones in a thick-walled gallbladder and a tumorous mass of the liver. Considering the patient's history of alcoholic liver cirrhosis this was thought to be a hepatocellular carcinoma. The patient was then referred to our surgical department for further evaluation.

**Figure 1:**
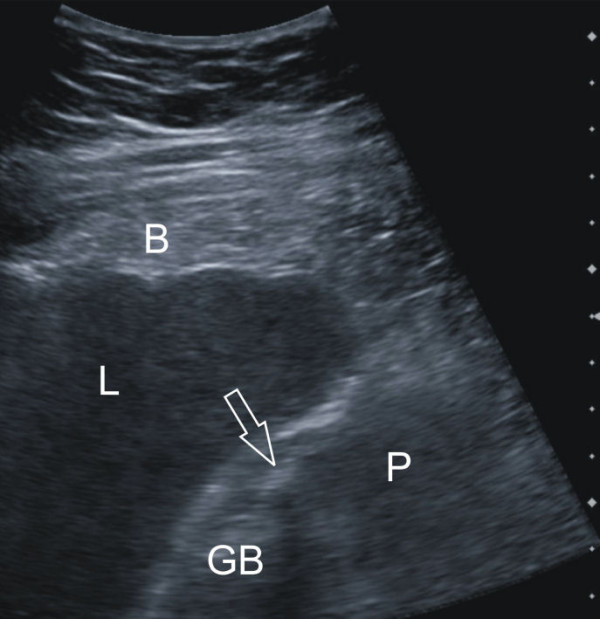
**Sonography of the abdomen**. This was performed after admission to our surgical department. Because of the lack of dorsal ultrasound reinforcement, the mass (P) surrounding the gallbladder (GB) was considered to be blood, pus or less likely tumorous soft tissue, not ascites. The transparent arrow indicates a stone.

On admission he had no elevated temperature (35.9°C), was hypotensive (80/40 mmHg) and tachycardic (120-140 beats/minute). He complained of upper abdominal pain persisting for about twenty-four hours. He had been treated with analgesics in the other clinic but with no relief. The physical examination confirmed tenderness of the right upper quadrant with initial signs of peritoneal irritation. At this point the laboratory studies revealed a significantly elevated white cell count (25 G/L) but once again no other abnormalities. The urine analysis showed elevated urobilinogen levels (2.0 mg/L). Sonography was repeated and it revealed a 7 × 6 cm conglomerate tumor of the gallbladder suspected of being an empyema, blood or a gallbladder carcinoma. Ascites was noticed around the liver (Fig. 1).

**Figure 2:**
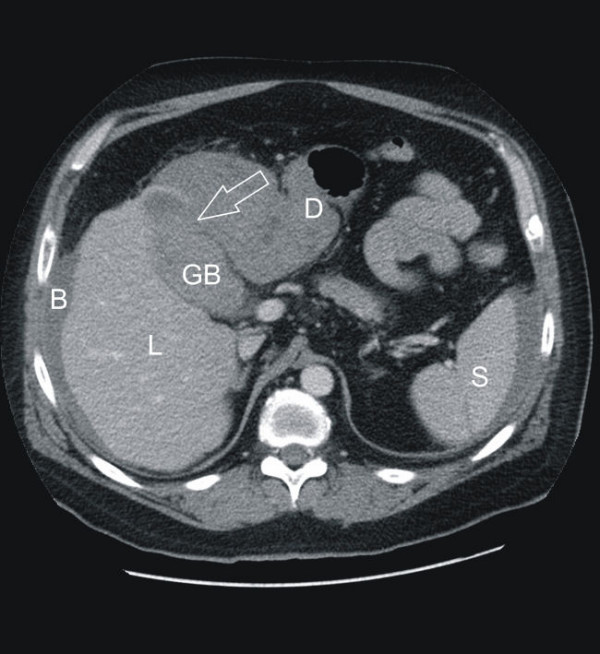
**Computed tomography (CT) of the abdomen (a: axial slice)**. L = liver, GB = gallbladder, D = duodenum, S = spleen, B = blood. The perforation site is indicated by the transparent arrow.

**Figure 3:**
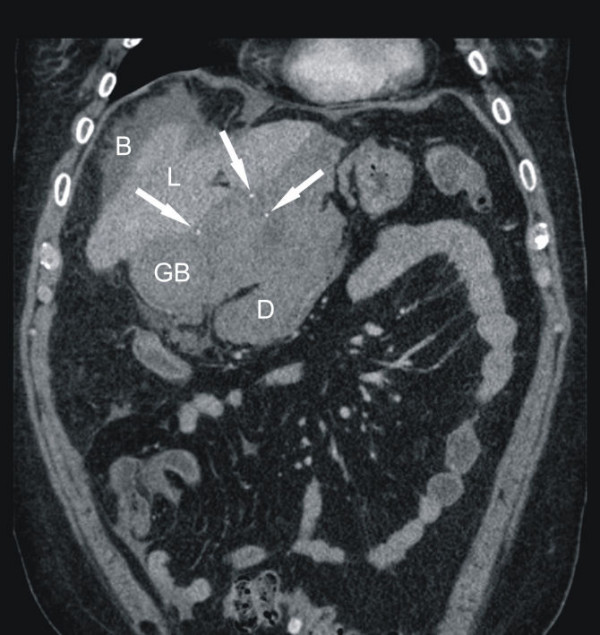
**Computed tomography (CT) of the abdomen (coronal reformation)**. L = liver, GB = gallbladder, D = duodenum, S = spleen, B = blood. Several calcified stones are appreciated outside the gallbladder (solid arrows in figure 2b). Notice also progredient hyperdense fluids surrounding liver and spleen (B), altogether making the diagnosis of free gallbladder perforation obvious.

The external CT was only available as nondiagnostic paper prints of axial slices using soft tissue windowing without both the possibility to perform attenuation measurements and the visualization in another plane or window. For this reason it was decided to repeat the CT scan around ten hours after the first one with a 64-row Scanner. The second scan confirmed the presence of the predescribed pericholecystic mass consistent with blood or pus (55 Hounsfield units). The diagnosis of a perforation was obvious since the gallstones were now found outside the gallbladder (Fig. 2 and 3).

**Figure 4:**
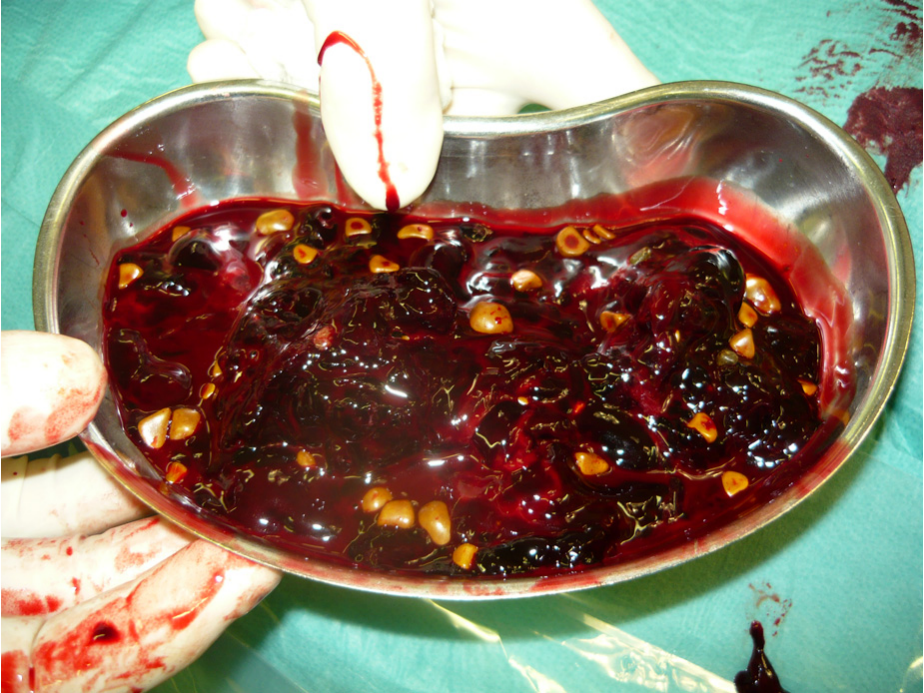
**Intraoperative picture of the fluid from the patient's abdomen containing stones and clotted blood**.

**Figure 5:**
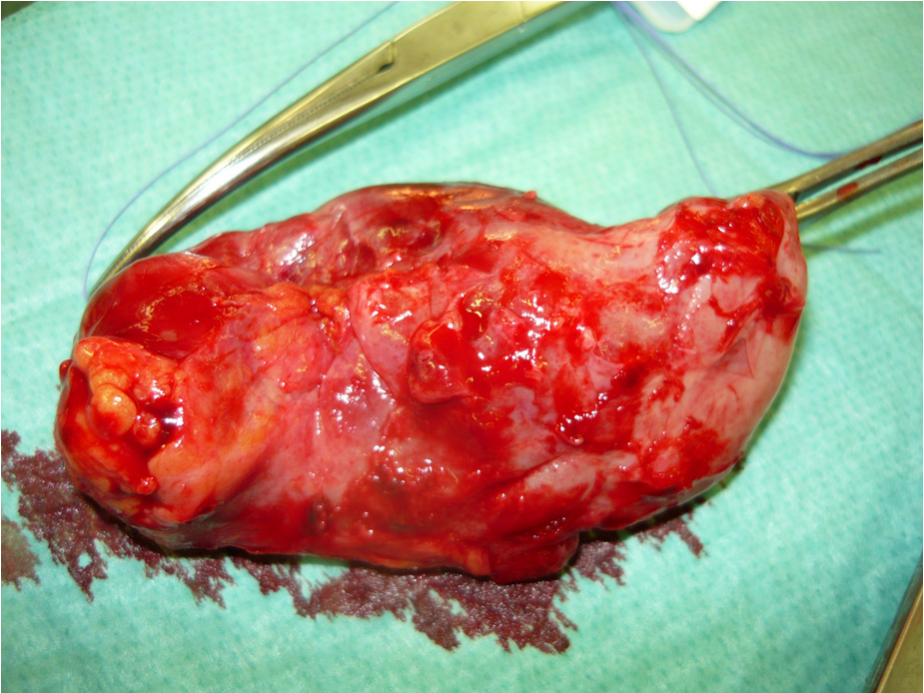
**Intraoperative picture: the perforated gallbladder**.

The patient received parenteral fluids, analgesics and antibiotics. Two hours later he was taken to the operating room for open cholecystectomy. A large quantity of blood and stones (Fig. 4) as well as the gallbladder which was perforated at the fundus site were removed (Fig. 5). After haemostasis and lavage, an Easy-Flow-Drain was placed in situ and the abdomen was closed. The patient was admitted to the ICU postoperatively and was transferred to a surgical ward twenty-four hours later. He recovered well and was discharged one week later.

## Discussion

Perforation can develop early in the course of acute cholecystitis (one or two days) or it may even occur several weeks after onset. The most common site of perforation is the fundus, presumably because of its poor blood supply (60% of the cases in the study of Derici et al. [1]). If the perforation locates at the fundus, it is less likely to be covered by the omentum thus bile and stones are likely to drain into the peritoneal space, as it happened in this case. If the perforation occurs at the isthmus or ductus, it is more easily sealed off by the omentum or the intestines and the condition remains limited to the right upper quadrant with formation of local inflammation and pericholecystic fluid.

Since there are no classical symptoms and signs of perforation diagnosis is challenging. Right upper quadrant pain, palpable right upper quadrant tenderness or high fever may indicate an acute onset. On the other hand patients may also show weakness, malaise and a palpable right upper quadrant mass, mimicking a malignacy. As most of these features are also present in acute cholecystitis, it is difficult to discriminate clinically between patients with perforated gallbladder and those with uncomplicated acute cholecystitis. A sudden decrease in pain intensity caused by the relief of high intracholecystic pressure might herald the perforation according to Chen et al. [4]. Gore et al [5] suggest that perforation and abscess formation should be suspected in those patients with acute cholecystitis who suddenly become toxic and whose clinical condition is found to deteriorate rapidly. Tsai et al. [6] propose to consider gallbladder perforation particularly in patients who are older than 70 years and have a high segmented neutrophil count (>80%).

Also the sonographic appearances of gallbladder perforation are diverse and nonspecific. They include wall thickening (>3 mm), distension (largest diameter >3.5-4.0 cm), gallstones, coarse intracholecystic echogenic debris and bile duct dilatation. Distention of the gallbladder and edema of its wall may be the earliest detectable signs of imminent perforation. The 'hole sign' (a defect in the gallbladder wall) is the most specific finding [7]. An intrahepatic perforation is suggested by the presence of a liver abscess with direct continuity into the gallbladder or containing echogenic stones in the absence of a pericholecystic abscess. Also the impossibility to visualize the gallbladder in the presence of a liver abscess is highly suggestive of an intrahepatic perforation[8].

Although ultrasound remains the preferred initial examination for evaluation of suspected gallbladder perforation, unfortunately it often fails to demonstrate the perforation because of increased intestinal gas and pain. In the current case the blood in and around the gallbladder led to a misinterpretation of the sonographic image. In contrast, CT imaging is the most sensitive tool to diagnose gallbladder perforation [7,8]. CT scan findings can be divided into primary gallbladder changes, pericholecystic changes and findings of extra-gallbladder organs. Primary gallbladder changes include wall thickening, wall enhancement, wall defect, intramural abscess, intramural gas, mural hemorrhage, presence of gallstones, common bile duct stones or cystic duct stones, intraluminal membrane and intraluminal gas. Pericholecystic changes include pericholecystic fat stranding, pericholecystic fluid collection, pericholecystic abscess or biloma formation and presence of extraluminal stones. Findings in organs other than the gallbladder consist of pericholecystic liver enhancement, liver abscess, portal vein thrombosis, reactive mural thickening of adjacent hollow organ (hepatic flexure of colon and duodenum), presence of lymph nodes, intraperitoneal free air, ascites, ileus and Mirizzi syndrome [8]. The gallbladder perforation signs can be divided into direct and indirect signs: the demonstration of either calculi outside the gallbladder or a ruptured segment of the gallbladder wall are direct indicators according to Pedrosa et al [9]. Indirect indicators include the presence of an abscess outside the gallbladder and the presence of gallstones together with thickening of the gallbladder wall. In the current case the best diagnostic clue of the first CT scan was the misinterpreted hyperdense fluid surrounding the gallbladder, the liver and the spleen. Measurement of the attenuation values should have led to the diagnosis of blood in as well as around the gallbladder, supporting the correct diagnosis.

Early diagnosis and surgical intervention are the key factors to decrease mortality and morbidity in the management of acute cholecystitis with gallbladder perforation. Both have significantly improved over the last few decades. This is partly due to shifting treatment paradigms in recent years with a larger number of cholecystectomies being performed for symptomatic cholelithiasis compared to the past but also the result of better diagnostic possibilities through the use of CT scans.

Despite this development, the management of cirrhotic patients with gallbladder perforation - as in this case - remains a greater challenge. Edema of the gallbladder wall, leukopenia caused by hypersplenism and the presence of ascites that predispose to spontaneous bacterial peritonitis make the diagnosis of gallbladder perforation more difficult than in the general population [10]. In addition cirrhotic patients have a higher rate of intraoperative and postoperative complications. In Child-Pugh A and B cirrhotic patients who undergo laparoscopic cholecystectomy, the overall mortality does not statistically differ from that of the general population. On the other hand the overall morbidity rate was found to be 21% compared with 8% for the general population in the meta-analysis of Silva et al. [11]. In patients with Child-Pugh C cirrhosis the mortality rate after cholecystectomy for acute cholecystitis is as high as 17%-25% [12]. For this reason less invasive treatments such as percutaneous gallbladder aspiration and cholecystostomy drainage have been recommended for advanced liver cirrhosis [10,13]. The 49-year-old man of the current case had Child-Pugh A alcoholic liver cirrhosis. He underwent open cholecystectomy and had no postoperative complications.

In conclusion gallbladder perforation is a rare but very serious condition and should be diagnosed and treated as soon as possible to decrease morbidity and mortality. The most important diagnostic tool is an early CT scan, followed by cholecystectomy on an emergency basis.

## Competing interests

The authors declare that they have no competing interests.

## Authors' contributions

CC (surgical resident) and MS (surgical attending) examined the patient in the ER, SW (radiology attending) diagnosed gallbladder perforation. CC and MS operated on the patient and took the photographs. All authors read and approved the final manuscript.
